# Procalcitonin levels in acute exacerbation of COPD admitted in ICU: a prospective cohort study

**DOI:** 10.1186/1471-2334-8-145

**Published:** 2008-10-23

**Authors:** Cédric Daubin, Jean-Jacques Parienti, Astrid Vabret, Michel Ramakers, Sabine Fradin, Nicolas Terzi, François Freymuth, Pierre Charbonneau, Damien du Cheyron

**Affiliations:** 1 Department of Medical Intensive Care, Caen University Hospital, 14033 Caen Cedex, France; 2 Department of Infectious Diseases and Biostatistics and Clinical Research, Caen University Hospital, 14033 Caen Cedex, France; 3 Department of Virology, Caen University Hospital, 14033 Caen Cedex, France; 4 Department of Biochemistry, Caen University Hospital, 14033 Caen Cedex, France

## Abstract

**Background:**

Antibiotics are recommended for severe acute exacerbation of chronic obstructive pulmonary disease (AECOPD) admitted to intensive care units (ICU). Serum procalcitonin (PCT) could be a useful tool for selecting patients with a lower probability of developing bacterial infection, but its measurement has not been investigated in this population.

**Methods:**

We conducted a single center prospective cohort study in consecutive COPD patients admitted to the ICU for AECOPD between September 2005 and September 2006. Sputum samples or tracheal aspirates were tested for the presence of bacteria and viruses. PCT levels were measured at the time of admittance, six hours, and 24 hours using a sensitive immunoassay.

**Results:**

Thirty nine AECOPD patients were included, 31 of which (79%) required a ventilator support at admission. The median [25%–75% interquartile range] PCT level, assessed in 35/39 patients, was: 0.096 μg/L [IQR, 0.065 to 0.178] at the time of admission, 0.113 μg/L [IQR, 0.074 to 0.548] at six hours, and 0.137 μg/L [IQR, 0.088 to 0.252] at 24 hours. The highest PCT (PCTmax) levels were less than 0.1 μg/L in 14/35 (40%) patients and more than 0.25 μg/L in 10/35 (29%) patients, suggesting low and high probability of bacterial infection, respectively. Five species of bacteria and nine species of viruses were detected in 12/39 (31%) patients. Among the four patients positive for *Pseudomonas aeruginosa*, one had a PCTmax less than 0.25 μg/L and three had a PCTmax less than 0.1 μg/L. The one patient positive for *Haemophilus influenzae *had a PCTmax more than 0.25 μg/L. The presence or absence of viruses did not influence PCT at time of admission (0.068 vs 0.098 μg/L respectively, *P *= 0.80).

**Conclusion:**

The likelihood of bacterial infection is low among COPD patients admitted to ICU for AECOPD (40% with PCT < 0.1 μg/L) suggesting a possible inappropriate use of antibiotics. Further studies are necessary to assess the impact of a procalcitonin-based therapeutic strategy in critically ill COPD patients.

## Introduction

Acute exacerbations are a leading cause of severe respiratory failure in chronic obstructive pulmonary disease (COPD) patients [1]. In this setting, the use of antibiotics is recommended by the recent guidelines of the European Respiratory Society (ERS), and French Consensus Conference [2,3]. However, bacteria are isolated from the respiratory tract of only approximately 50% of patients with severe acute exacerbation of COPD (AECOPD) [4-6]. Whether this finding represents colonization or infection is controversial [7-9]. In contrast, a high prevalence of respiratory viruses has been reported in severe AECOPD requiring ventilation [10,11].

In this context, a rapid, specific test to identify lower respiratory bacterial infections would be a major advancement, limiting the inappropriate use of antibiotics which is considered to be a main cause of the spread of antibiotic-resistant bacteria [12,13]. Serum procalcitonin levels are considered to be one of the best biomarkers for predicting a bacterial infection [14]. The level of circulating procalcitonin is increased in severe bacterial infections, but remains fairly low in viral infection and non specific inflammatory diseases [15,16]. In addition, procalcitonin-based therapeutic strategies have been shown to substantially and safely reduce antibiotic use in patients with no severe lower respiratory tract infections [17] or acute exacerbation of COPD [18]. However, studies that specifically assess this marker in critically ill patients are scarce.

The aim of the current study was to assess procalcitonin levels in, and their relationship with viruses and bacteria in respiratory samples from COPD patients admitted to the ICU for AECOPD.

## Materials and methods

### Patients

COPD patients with suspected lower respiratory tract infection admitted to the medical intensive care unit of the University Hospital of Caen between September 2005 and September 2006 were assessed for eligibility. Only those with AECOPD were included in the analysis. Patients with the presence of infiltrates on chest radiographs taken at admission and suspected of pneumonia, or exhibiting evidence of other causes of respiratory failure, were not included in this study.

### Definition

We defined COPD according to the Global Initiative for Chronic Obstructive Lung Disease Guidelines (GOLD) 2005  as an FEV1/FVC ratio (forced expiratory volume in 1 second/functional vital capacity) of less than 70%, with severity categorized into mild (FEV1 ≥ 80% of predicted), moderate (FEV1 ≥ 50% to < 80%), severe (FEV1 ≥ 30% to < 50%) and very severe (FEV1 < 30%). An AECOPD was defined as "a sustained worsening of patient's condition, from stable state and beyond normal day-to-day variations, that is acute in onset and necessitates a change in regular medication in a patient with underlying COPD" [19].

AECOPD was considered bacteriologically confirmed in the presence of a positive Gram stain of respiratory samples, a pathogen concentration greater than 10^5^cfu/mL in tracheobronchial aspirations, or a blood culture revealing a bacterial pathogen in the absence of an extrapulmonary focus [4]. A culture, indirect immunofluorescence assay (IFA) or polymerase chain reaction (PCR) assay positive for viruses was considered evidence for a viral pathogen.

### Study design

The protocol of this prospective cohort study was submitted to the local ethics committee. The ethical board decided that approval was not necessary given the observational nature of our study. Thus, according to French legislation at the time of the study, no informed consent was obtained from the patients. Upon admission to the ICU, baseline assessment included the severity of COPD according to GOLD criteria; home oxygen and home non-invasive ventilation treatment; chronic steroid therapy; comorbidities such as cigarette smoking, chronic alcohol abuse, obesity, diabetes mellitus, or chronic cardiovascular diseases; use of antibiotic therapy and oral steroids for exacerbations of COPD during the previous 30-day period; use of antibiotics within 24 hours of ICU admission; physical examination; chest radiography; and routine blood tests including c-reactive protein (CRP) at admission (CRP-H0) and 24 hours later (CRP-H24). In addition, scoring of disease severity the first day in ICU was assessed by the Simplified Acute Physiology Score type II (SAPS II) [20], the Acute Physiology and Chronic Health Evaluation (APACHE II) score[21], and the Logistic Organ Dysfunction (LOD) score [22]. All patients were treated according to the recommendations of the French Consensus Conference [3].

### Measurement of serum procalcitonin

All blood samples were centrifuged, decanted, aliquoted, and frozen at -80°C until analyzed at the end of the study period. PCT was measured using a sensitive immunoassay (Kryptor PCT, Brahms, Hennigsdorf, Germany) with a functional assay sensitivity of 0.06 μg/L, about fourfold above mean normal levels [23]. The circulating levels of procalcitonin were sequentially assessed at ICU admission (PCT-H0), after six hours (PCT-H6), and twenty fours (PCT-H24) hours. Patients were classified into three groups based on the probability of bacterial infection according to the highest procalcitonin level measured (PCTmax), as previously reported [17,18]: group1 PCTmax < 0.1 μg/L indicating an absence of infection, group 2 PCTmax > 0.1 and < 0.25 μg/L indicating a possible infection and group 3 PCTmax > 0.25 μg/L indicating the presence of infection.

### Microbiological assessment

Upon enrollment, spontaneously expectorated sputum samples or tracheal aspirates were obtained for Gram staining and cultures. Bacterial isolation and identification was performed with the use of standard techniques. Sputum or tracheal aspirates were bacteriologically processed if less than 1% of the observed field contained squamous epithelial cells and more than 25 neutrophils were observed [24]. A serological diagnostic for antibodies to *Legionella pneumophila *was also performed by indirect immunofluorescence, associated with a detection of *Legionella pneumophila *serogroup 1 antigen in urine samples. In addition, nasal swab or tracheobronchial aspiration was performed in all patients and tested for viruses. Details of the viral detection methods including culture, indirect immunofluorescence assay (IFA), and molecular methods (PCR or RT-PCR), are published elsewhere [11,25-27]. We tested for the following viruses: parainfluenza virus (PIV) 1,2,3, and 4; influenza virus A,B, and C; respiratory syncytial virus (RSV); metapneumovirus (hMPV); rhinovirus (RV); coronavirus 229E and OC43; and, adenovirus (AdV). *Chlamydia pneumoniae *and *Mycoplasma pneumoniae *were also detected by PCR assay.

### Statistical analysis

Quantitative and qualitative data were expressed as means (+/- SD), or median [25%–75% interquartile range] and percentage (with their 95% CI), respectively. Categorical variables were compared using the chi-square or Fischer's exact test as appropriate. Quantitative variables were compared using the Student *t*-test or the Mann-Whitney non parametric test as appropriate. Confidence intervals of percentages were based on normal approximation. The level of significance was set at 0.05 and all tests were two-sided. We used EPI-INFO version 6.04 dfr (EPI-INFO, CDC, Atlanta, GA) for data collection, and EPI-INFO and SAS version 9.1 (SAS Institute Inc, Cary, NC) for data analysis.

## Results

### Baseline characteristics

**Figure 1:**
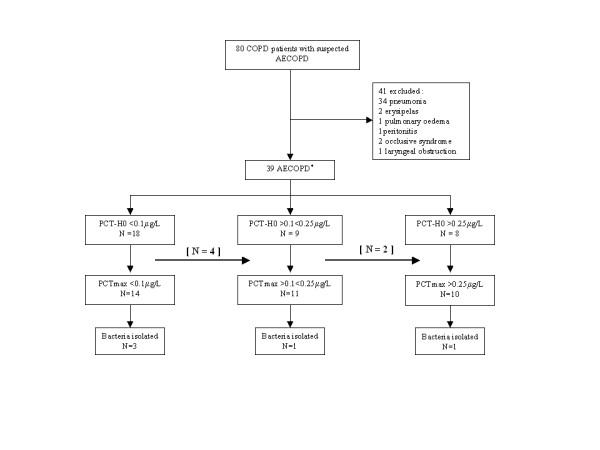
**The flow of the study measuring procalcitonin in COPD patients**. * Procalcitonin (PCT) levels were not assessed in 4 patients. ** The arrow indicate cross-over between groups.

During the period of the study, 80 COPD patients with suspected lower respiratory tract infection were admitted to the ICU, 39 of which had AECOPD (Figure 1). Baseline characteristics of the AECOPD patients are shown Table 1. Twenty nine patients (74%) had severe or very severe COPD. Twenty seven (69%) received home oxygen or home non-invasive ventilation. Use of antibiotic therapy or oral steroids for exacerbations of COPD during the previous 30-day period was reported in 13 and 11 patients, respectively. Nine patients had received antibiotics within 24 hours of ICU admission. The following treatments were given at admission: non invasive ventilation (NIV) in 27 (69%) patients; mechanical ventilation in 6 (15%) patients, 2 of whom after NIV failure; antibiotics in 33 (85%) patients; systemic steroids in 30 (77%) patients; and a β_2_-agonist with inhaled steroids in all patients.

**Table 1:** Baseline characteristics of all patients and according to the maximum procalcitonin levels measured (PCTmax)

**Characteristics**	AECOPD n = 39*	**PCTmax<0.1 n = 14**	**0.1<PCTmax<0.25 n = 11**	**PCTmax>0.25 n = 10**
Age, yr	62 ± 15	54 ± 16	71 ± 10	66 ± 7
Male sex, no.(%)	26(67)	7(50)	8(73)	9(90)
				
SAPS II score^¶^	30 [23–35]	25 [18–30]	31 [26–33]	39 [28–40]
APACHE II score^¶¶^	18 [12–23]	13 [9–23]	18 [16–22]	19 [18–26]
LOD score^¶¶¶^	4 [2–5]	3 [2–4]	4 [2–4]	5 [3–8]
				
Comorbidities, no. (%)				
Current smokers,	11(28)	4(29)	4(36)	3(30)
Chronic alcohol abuse	8(20)	3(21)	2(18)	2(20)
Obesity	8(20)	2(14)	4(36)	2(20)
Coronary artery disease	4(10)	1(7)	0	3(30)
Hypertensive heart disease	15(38)	4(29)	4(36)	5(50)
Congestive heart disease	2(5)	0	1(9)	0
Diabetes mellitus	13(33)	6(42)	4(36)	3(30)
				
Chronic Pseudomonas colonization, no. (%)	5(13)	3(21)	0	2(20)
				
Severity of COPD^¶¶¶¶^, no. (%)				
GOLD stage I (mild)	7(18)	4(29)	1(9)	0
GOLD stage II (moderate)	3(8)	1(7)	2(18)	0
GOLD stage III (sever)	3(8)	0	2(18)	1(10)
GOLD stage IV (very sever)	26(67)	9(64)	6(54)	9(90)
Home oxygen, no. (%)	22(56)	8(57)	5(45)	8(80)
Home non-invasive ventilation, no. (%)	5(13)	2(14)	0	2(20)
Oral or inhaled steroid, no. (%)	23(59)	8(57)	7(64)	8(80)
				
Examination at ICU admission, no. (%)				
Dyspnea	39(100)	14(100)	11(100)	10
Cough	17(44)	5(35)	8(73)	4(40)
Sputum	15(33)	5(36)	5(45)	5(50)
Rales	4(10)	1(7)	1(9)	2(20)
Wheezing	30(77)	11(100)	10(90)	7(70)
Body temperature,°C	37.0 ± 0.8	36.7 ± 0.9	37.2 ± 0.5	37.2 ± 0.8
Leucocytes count ((×10^9^/L)	11.5 ± 4.3	9.3 ± 4.1	11.2 ± 4.6	14.2 ± 2.9
CRP-H0	19 [9–60]	13 [8–22]	15 [5–66]	43 [11–156]
CRP-H24	21 [11–34]	21 [11–24]	17 [9–32]	78 [11–106]

Values are given as No.(%), median [25%–75% interquartile range], or mean ± standard deviation

* Levels of procalcitonin (PCT) were assessed in 35/39 patients. PCT is given in μg/L

¶ *P = 0.005 *for comparing PCTmax > 0.25 μg/L versus PCTmax < 0.25 μg/L; ¶¶ *P = 0.08 *for comparing PCTmax > 0.25 μg/L versus PCTmax < 0.25 μg/L; ¶¶¶ *P = 0.018 *for comparing PCTmax > 0.25 μg/L versus PCTmax < 0.25 μg/L; ¶¶¶¶ *P = 0.07 *for comparing PCTmax > 0.25 μg/L versus PCTmax < 0.25 μg/L

### Laboratory measurements

**Figure 2:**
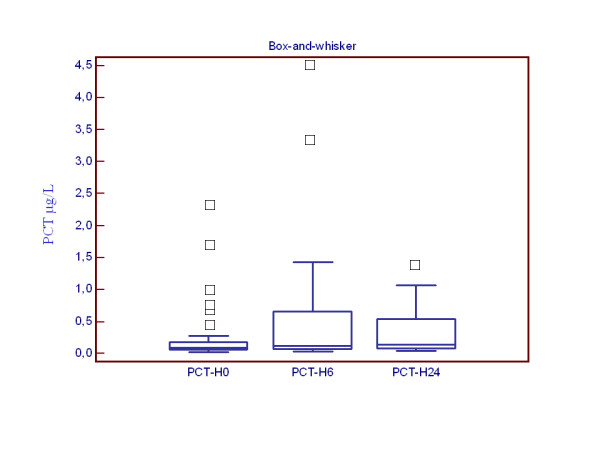
**Box-and-whisker plots representing PCT levels measured at various time**. On the horizontal axis, and for each boxplot, the legend corresponds to the time of PCT measurement. On the vertical axis, the number corresponds to the PCT levels (μg/L). Each box denotes the middle 50% of the data measured at that time. The lower and upper ends of the box denote the 25^th ^and 75^th ^percentile, respectively. The solid black horizontal lines through each box denote the median of the distribution. The vertical solid black lines (the "whisker") reach out to the 1.5 interquartile range. Circles above the whisker denote individual external observations.

The circulating levels of procalcitonin are shown in Figure 2. The median [25%–75% interquartile range] procalcitonin levels at admission (PCT-H0) were 0.096 μg/L [0.065–0.178], PCT-H6 was 0.113 μg/L [0.074–0.548], and PCT-H24 was 0.137 μg/L [0.088–0.252]. Procalcitonin levels were not different in patients who had received antibiotics in the month or the 24 hours prior to ICU admission compared to antibiotic-naive patients (PCT-H0 0.071 μg/L [0.056–0.189] vs 0.099 μg/L [0.065–0.178], *P *= 0.36 and PCT-H0 0.081 μg/L [0.071–0.397] vs 0.103 μg/L [0.062–0.178], *P *= 0.93, respectively). There was no association between PCT-H0 levels and the presence or absence of sputum (PCT-H0 0.106 μg/L [0.072–0.231] vs 0.085 μg/L [0.062–0.163], *P *= 0.65, cough (PCT-H0 0.083 μg/L [0.068–0.162] vs 0.103 μg/L [0.062–0.695], *P *= 0.57, wheezing (PCT-H0 0.083 μg/L [0.057–0.178] vs 0.103 μg/L [0.075–0.695], *P *= 0.29, and fever (ie temperature > 38°C) (PCT-H0 0.081 μg/L [0.068–0.103] vs 0.098 μg/L [0.059–0.183], *P *= 0.92). The PCTmax was < 0.1 μg/L in 14/35 patients (40%), between 0.1 and 0.25 μg/L in 11/35 patients (31%), and > 0.25 μg/L in 10/35 patients (29%). There was no association between the PCTmax levels and the severity of COPD (*P *= 0.07). Patients with PCTmax > 0.25 μg/L were more critically ill: SAPS II 39 [28–40] vs 27 [24–31] among patients with PCTmax ≤ 0.25 μg/L, *P *= 0.005 ; and LOD 5 [3–8] vs 3 [2–4] among patients with PCTmax ≤ 0.25 μg/L, *P *= 0.018. The median CRP level at admission (CRP-H0) was 19 mg/L [9–60] and CRP-H24 was 21 mg/L [11–34] and was not significantly higher among those with a PCTmax > 0.25 μg/L (CRP-H0 43 μg/L [11–156] vs 14 [8–24], *P *= 0.12 and CRP-H24 78 μg/L [11–106] vs 20 [11–26], *P *= 0.17).

### Bacteria and viral findings

Samples were taken at admission in the form of sputum, tracheobronchial aspirates, or nasal swabs for bacteria and viral studies in 29 (75%) and 34 (87%) patients, respectively, but failed because of technical problems in 10 and 5 patients, respectively. Additionally, hemocultures and *Legionella *diagnostic tests were performed in 37 (95%) and 38 (97%) patients, respectively. According to our definition, 12 (31%) patients had AECOPD microbiologically confirmed. Five species of bacteria (4 *Pseudomonas aeruginosa*, 1 *Haemophilus influenzae*) and 9 species of viruses (6 rhinovirus, 1 human metapneumovirus, 1 parainfluenza 1 and 1 parinfluenza 3) were detected. A co-infection was detected in 2 cases (rhinovirus and *Pseudomonas aeruginosa*, rhinovirus and *Haemophilus influenzae*). PCT-H0 levels did not differ between patients with or without AECOPD bacteriologically confirmed (PCT-H0 0.081 μg/L [0.062–0.189] vs 0.098 μg/L [0.065–0.170], *P *= 0.75). The four patients with *Pseudomonas aeruginosa *had a PCTmax < 0.25 μg/L. Three of them were known to be chronically colonized by this germ and had a PCTmax < 0.1 μg/L. A positive-virus respiratory sample did not influence PCT levels at admission (PCT-H0 0.068 μg/L [0.062–0.695] vs 0.098 μg/L [I0.065–0.163], *P *= 0.80).

### Clinical outcome

During their ICU stay, non-invasive ventilation or mechanical ventilation were performed in 31 patients (79%). The mean length of non-invasive ventilation, mechanical ventilation, and ICU stay without ventilation in days were 4.33 ± 4.54, 2.10 ± 5.55, and 2.13 ± 1.89, respectively. There was no correlation between these findings and procalcitonin levels (data not shown). The mean length of ICU stay was 9 ± 7 days. One patient developed a ventilator-associated pneumonia. He died of COPD-related respiratory failure seven days after ICU discharge. In this patient, the PCT levels increased during the first 24 hours (PCT-H0 0.695 μg/L to PCT-H24 1.388 μg/L). No patient developed septic shock. Thirty five patients were discharged from the hospital. Twelve of these patients (34%) were subsequently hospitalised for AECOPD within 6 months. PCT-H0 and PCTmax were not predictive of recurrence (data not shown). Four patients died, three of COPD-related respiratory failure and one of pneumothorax complicated with cardiac arrest. All had very severe COPD and a PCTmax > 0.25 μg/L. In univariate analysis, a PCT level > 0.25 μg/L was associated with mortality (4/10 vs 0/25 deaths among those with a PCT < 0.25 μg/L, *P *< 0.006).

## Discussion

The present study reports procalcitonin levels in critically ill COPD patients admitted for AECOPD with severe conditions according to the Global Initiative for Chronic Obstructive Lung Disease criteria. In agreement with previous reports [17,18], PCT levels (< 0.1 μg/L) could indicate an absence of infection in approximately 40% of patients. In this sub-group, no bacteria, were detected in systematic screening, except *Pseudomonas aeruginosa *in three patients known to be chronically colonized by this pathogen. These preliminary results may have important implications for investigating procalcitonin-based antibiotic strategies in severe AECOPD.

Our findings differ from previous studies assessing the microbiological pattern of AECOPD [4,6,10,28-30], which reported a higher prevalence of bacteria, varying from 23% [10] to 72% [4]. Differences in inclusion criteria and diagnostic tests in an absence of respiratory samples for bacteria detection in a quarter of our patients may explain this difference. Our rates of virus-positive respiratory samples (23%) was consistent with the 22 to 46% prevalence of respiratory viral infection observed in recent prospective studies using molecular methods and focusing on COPD patients [10,11,30-32].

We found a PCT level of 0.096 μg/L [0.065–0.178] in COPD patients admitted to the ICU for severe AECOPD, consistent with previous studies in this setting (0.096 μg/L [0.070–0.200] [18]; 0.088 μg/L [0.053–0.161] [33]). However, in these two large prospective cohorts focusing on hospitalized AECOPD, less than 10% of patients were admitted to ICU. According to Stolz et al. [18], PCT levels were not different between patients pre-treated with antibiotics and antibiotic-naive patients, and no association was found between PCT levels and clinical symptoms or AECOPD bacteriologically confirmed. In addition, the proportion of patients with particular levels of PCT was in accordance with this previous report, suggesting that a large fraction of patients with low PCT levels (PCTmax < 0.1 μg/L) have a low likelihood of bacterial infection. In this sub-group, only one pathogen, *Pseudomonas aeruginosa*, was detected in three patients known to be chronically colonized by this germ. Whether this finding represents colonization or infection remains controversial [7,32,34,35]. This could explain why PCT measurements failed to identify 3 from 5 patients with AECOPD bacteriologically documented in our study. In one case, rhinovirus was isolated in association with *Pseudomonas aeruginosa*, and was considered as the cause of exacerbation. Moreover, we reported that a lower respiratory tract viral infection did not significantly influence PCT levels in this setting. In addition, despite our small sample size, we were able to demonstrate a significant impact of PCT (PCT > 0.25 μg/L in this cohort) in predicting ICU mortality, as previously reported in studies focusing on septic shock [36] and ventilator-associated pneumonia [37].

This study had some limitations. The monocentric design of the study and, the relatively small sample size, as well as the fact that sputum could not be taken in 10 patients (25%) for bacteria detection, may limit the interpretation and relevance of our data. However, PCT assessment was performed in 90% of patients using technology recognized as the most sensitive, and our findings are consistent with those of previous studies [17,18]. For this reason, we believe that this study adds useful information about PCT levels in severe AECOPD requiring an ICU stay and the likelihood of bacterial infection.

The results reported here may have important implications for the design of a randomized controlled trial testing procalcitonin-based antibiotic strategies in severe AECOPD. It has been demonstrated antibiotics have a marginal efficacy in the treatment of AECOPD, except among patients with evidence of bacterial infection or severe exacerbation [38]. Less than 50% of severe AECOPD may be attributed to bacteria, suggesting the potential for excessive antibiotic use in this setting [39]. Therefore, serum procalcitonin level may be considered as a useful tool for predicting bacterial infection, and may prove useful for selecting patients with a lower probability of bacterial infection and limit the inappropriate use of antibiotics, specifically in the ICU setting where antibiotic use and the emergence of antimicrobial resistance are highly prevalent [40]. In this context, we speculate that antibiotic use in the subgroup of severe AECOPD with lower PCT (< 0.1 μg/L), could be reduced as previously reported in studies assessing a procalcitonin-based antibiotic strategy in non-severe lower respiratory tract infection [17,18].

## Conclusion

Our results support that 40% of patients admitted to ICU for AECOPD have a low likelihood of bacterial infection and correlates with a PCT less than 0.1 μg/L, suggesting a possible inappropriate use of antibiotics. However, further studies are necessary: to assess the short-term effect of a procalcitonin-based therapeutic strategy in critically ill COPD patients in ICU and secondly, to address the role of antimicrobial agents in COPD patients in the long-term, particularly in patients with low PCT levels and bacteria-positive sputum.

## Abbreviations

AECOPD: acute exacerbation of chronic obstructive pulmonary disease; APACHE II: acute physiology and chronic health evaluation type II; MICU: medical intensive care unit; IFA: indirect immunofluorescence assay; LOD: logistic organ dysfunction system; PCR: polymerase chain reaction; PCT: procalcitonine; SAPS II: simplified acute physiology score II

## Competing interests

The authors declare that they have no competing interests.

## Authors' contributions

CD and MR initiated the study, the design, and the experimental protocol. AV and FF performed the virologic assessments. SF performed the PCT measurements. CD and JJP performed the statistical analysis and were involved in the interpretation of the results. CD wrote the manuscript, and JJP, MR, and DDC helped to draft the manuscript. DDC, MR, NT, and PC contributed to the conception and design of the study and revision of the manuscript. All authors read and approved the final manuscript.

## Pre-publication history

The pre-publication history for this paper can be accessed here:


